# Variation of chemosensory receptor content of *Campylobacter jejuni* strains and modulation of receptor gene expression under different *in vivo* and *in vitro* growth conditions

**DOI:** 10.1186/1471-2180-12-128

**Published:** 2012-06-29

**Authors:** Christopher J Day, Lauren E Hartley-Tassell, Lucy K Shewell, Rebecca M King, Greg Tram, Serena K Day, Evgeny A Semchenko, Victoria Korolik

**Affiliations:** 1 Institute for Glycomics, Griffith University Gold Coast Campus, Griffith University, Griffith, Australia; 2 Department of Microbiology, Pathology Queensland, Gold Coast Hospital, Southport, Australia

**Keywords:** Chemotaxis receptor, Campylobacter, Transducer-like proteins

## Abstract

**Background:**

Chemotaxis is crucial for the colonisation/infection of hosts with *Campylobacter jejuni.* Central to chemotaxis are the group A chemotaxis genes that are responsible for sensing the external environment. The distribution of group A chemoreceptor genes, as found in the *C. jejuni* sequenced strains, *tlp1-4, 7, 10* and *11* were determined in 33 clinical human and avian isolates.

**Results:**

Group A *tlp* gene content varied among the strains with genes encoding *tlp1* (aspartate receptor, *ccaA*) and *tlp7* present in all strains tested, where as *tlp11* was present in only one of our international collection clinical isolates, *C. jejuni* 520, but was more prevalent (9/13) in the freshly isolated clinical stains from patients who required hospitalisation due to *C. jejuni* infection (GCH1-17). Relative expression levels of the group A *tlp* genes were also determined in *C. jejuni* reference strains NCTC 11168-GS, 11168-O and 81116 using cells grown *in vitro* at 37°C, 42°C and maintained at room temperature and with cells isolated directly from murine and avian hosts by immune magnetic separation without subsequent culture. Gene expression of *tlp* genes was varied based on strain, growth conditions and *in vivo* isolation source. *Tlp1,* although the most conserved, showed the lowest and most varied mRNA expression and protein production under laboratory conditions. *Tlp7* was highly expressed at most conditions tested, and gene expression was not influenced by the *tlp7* gene encoding a full length protein or one expressed as separate periplasmic and cytoplasmic domains.

**Conclusion:**

We have shown that chemosensory receptor set variation exists among *C. jejuni* strains, but is not dependent on the isolation source.

## Background

*Campylobacter jejuni* is a causative agent of acute bacterial gastroenteritis in humans, and is responsible for an estimated 500 million cases annually worldwide [1,2]. Although this bacterium poses a significant economic burden, little is known or understood about the mechanisms of pathogenicity. Some factors, however, have been ascertained to contribute toward the overall pathogenicity of the infecting strain such as chemotaxis, adherence to host cells and surface glycans including lipooligosaccharide [3].

Chemotaxis and motility have been implicated in the colonisation and virulence of many pathogenic bacteria such as *Escherichia coli, Salmonella enterica* serovar Typhimurium, as well as *C. jejuni*[3,4]. Homologues of the chemotactic pathway have been identified in *C. jejuni* NCTC 11168 and include ten putative chemotactic sensory receptors, Tlps, and two aerotaxis receptors [5]. The receptors are grouped according to their putative function as assigned by homology to known chemoreceptors of other organisms [5,6]. The group A consist of Tlp1, 2, 3, 4, 7 and 10, all of which contain distinct domains comprising of two transmembrane domains, a sensory domain and a highly conserved cytoplasmic domain [5]. Due to similarity to methyl-accepting chemotactic proteins from other bacterial species, group A Tlp receptors are thought likely to sense ligands external to the cell [5]. Only two of the group A Tlp proteins of *C. jejuni* have been characterised to date, the aspartate receptor, Tlp1 [7] and Tlp7 which binds to formic acid [8].

Recent analysis of full and partial sequenced strains of *C. jejuni* has shown diversity in the group A Tlp receptor set and indicated that Tlp1 was the only receptor universally represented in all sequenced strains of *C. jejuni*[6]. This high conservation can be explained by the fact that *tlp1* encodes the aspartate receptor for *C. jejuni*[7], aspartate being one of the carbon sources used in *C. jejuni* metabolism. The receptor set for 81116 was previously reported to be similar to that of 11168 genome sequenced strain, including that of Tlp7, which is represented as a “pseudogene”, however, Tlp7 is presumed to be a functional protein in strain HB93-13, as there is no stop codon to interrupt the sequence [6]. A recent study has shown that each portion of *tlp7* can be translated as separate proteins and still function in chemotaxis of this organism [8].

It has previously been suggested that receptor subset variation may be dependent on strain source or relative pathogenicity, since variance in the chemoreceptor subset has been shown for some uropathogenic strains of *E. coli*, which all lack the functional receptors Trg (ribose and galactose) and Tap (dipeptides) usually present within strains isolated from faecal material [9]. In *C. jejunitlp7* is the only receptor where this has been tested using strains from different sources. Zautner *et al.* (2011) showed that *dtlp7tlp7* encoded by two separate genes rather than a single transcript, was over-represented in bovine strains and underrepresented in human isolates [10].

In addition to 6 group A *tlp* genes encoded by *C. jejuni* 11168, a unique *tlp*, designated as Tlp11, was identified in some *C. jejuni* strains and was shown to share sequence similarity with TcpI, a chemoreceptor involved in stimulating the expression of the CT and TCP pathway of *Vibrio cholerae*[6]. It has yet to be established if Tlp11 exists in other *C. jejuni* isolates and whether it has a role in enhancing virulence or if it has an effect on the expression levels of the other group A *tlp* genes.

Although genome analysis has demonstrated which receptor sets are present in partially and fully-sequenced strains of *C. jejuni*, whether gene expression is conserved has yet to be elucidated. Here we report the variation in *C. jejuni* chemoreceptor gene subsets within the genomes of 33 *C. jejuni* strains, including NCTC 11168 -GS and –O, isolated from both avian and human hosts. *C. jejuni* 11168-GS is the non-colonising, non-invasive variant of NCTC 11168 with known decreases in virulence-associated phenotypes and with a number of point mutations when compared to the original isolate (11168-O) from which it was derived [11]. We also report receptor gene expression modulation *in vivo*, during colonisation of avian and mammalian hosts, and *in vitro* under varying growth conditions.

## Results

### Tlp gene content of different *C. jejuni* strains

Thirty-three strains of *C. jejuni* isolated from chicken and human hosts were analysed to elucidate which *tlp* genes were present in the genomes of these strains and if any relatedness to the isolation host could be ascertained. The identity of each group A Tlp receptor for all seven known group A *tlp* genes, *tlp1-4, 7, 10* and *11* in each of the 33 *C. jejuni* strains were determined by PCR amplification (Table 1). The *C. jejuni* strains tested appeared to possess varied sets of group A Tlp receptor genes, with six strains (*C. jejuni* 520, GCH3, 6, 10, 14 and 17) possessing all seven group A *tlp* genes (Table 1). *Tlp1* was present in all strains tested and is the only universally conserved *tlp* gene within the strains (Table 1). *Tlp7* was present in 31 of 33 strains, while, *tlp10* and *tlp3* were detected in 30 of 33 strains making them the next most conserved of the *tlp* genes (Table 1). The least representatively conserved *tlp* genes, other than *tlp11,* were *tlp2* and *tlp4* (Table 1).

**Table 1:** **Results of PCR amplification of *tlp *** **genes of *C. jejuni *** **strains isolated from both chickens and humans**

***C. jejuni strain***	**Tlp1**	**Tlp2**	**Tlp3**	**Tlp4**	**Tlp7**	**Tlp10**	**Tlp11**
**Chicken isolates**							
**008**	**+**	**-**	**+**	**+**	**+**^**P**^	**+**	**-**
**019**	**+**	**-**	**+**	**-**	**+**^**P**^	**+**	**-**
**108**	**+**	**-**	**+**	**+**	**+**^**P**^	**+**	**-**
**331**	**+**	**+**	**-**	**+**	**+**^**W**^	**+**	**-**
**434**	**+**	**-**	**+**	**+**	**+**^**W**^	**+**	**-**
**506**	**+**	**-**	**+**	**+**	**+**^**W**^	**-**	**-**
**913**	**+**	**+**	**+**	**-**	**+**^**W**^	**-**	**-**
**Human isolates Laboratory maintained**							
**173**	**+**	**-**	**+**	**+**	**+**^**W**^	**+**	**-**
**11168-GS**	**+**	**+**	**+**	**+**	**+**^**P**^	**+**	**-**
**11168-O**	**+**	**+**	**+**	**+**	**+**^**P**^	**+**	**-**
**351**	**+**	**+**	**+**	**-**	**+**^**W**^	**+**	**-**
**430**	**+**	**+**	**+**	**+**	**+**^**W**^	**+**	**-**
**435**	**+**	**+**	**+**	**+**	**+**^**W**^	**+**	**-**
**440**	**+**	**+**	**+**	**+**	**+**^**W**^	**+**	**-**
**520**	**+**	**+**	**+**	**+**	**+**^**W**^	**+**	**+**
**705**	**+**	**+**	**+**	**-**	**+**^**W**^	**+**	**-**
**8**	**+**	**-**	**+**	**+**	**+**^**W**^	**+**	**-**
**81116**	**+**	**+**	**+**	**+**	**+**^**W**^	**+**	**-**
**81–176**	**+**	**+**	**-**	**+**	**+**^**W**^	**+**	**-**
**93**	**+**	**+**	**+**	**+**	**+**^**W**^	**-**	**-**
**Human isolates Fresh clinical isolates**							
**GCH1**	**+**	**+**	**+**	**+**	**+**^**P**^	**+**	**-**
**GCH2**	**+**	**+**	**+**	**+**	**+**^**P**^	**+**	**-**
**GCH3**	**+**	**+**	**+**	**+**	**+**^**W**^	**+**	**+**
**GCH4**	**+**	**-**	**+**	**+**	**+**^**W**^	**+**	**+**
**GCH5**	**+**	**+**	**+**	**+**	**+**^**W**^	**+**	**-**
**GCH6**	**+**	**+**	**+**	**+**	**+**^**W**^	**+**	**+**
**GCH7**	**+**	**+**	**+**	**-**	**-**	**+**	**+**
**GCH9**	**+**	**+**	**+**	**+**	**+**^**P**^	**+**	**-**
**GCH10**	**+**	**+**	**+**	**+**	**+**^**W**^	**+**	**+**
**GCH11**	**+**	**-**	**-**	**-**	**+**^**W**^	**+**	**+**
**GCH14**	**+**	**+**	**+**	**+**	**+**^**W**^	**+**	**+**
**GCH15**	**+**	**+**	**+**	**-**	**-**	**+**	**+**
**GCH17**	**+**	**+**	**+**	**+**	**+**^**W**^	**+**	**+**

+ = Positive PCR product present in repeat experiments.

- = No product detected in repeat PCR amplifications.

+^P^ refers to the presence of tlp7 as two separately co-expressed genes.

+^W^ refers to a whole gene able to be translated into a complete protein product. Sequencing was performed in triplicate to ensure accuracy of the results.

### Sequencing results of *tlp7*

*Tlp7* is annoted as a “pseudogene” in *C. jejuni* 11168 though a recent study showed it is functional in strains that do not possess an uninterrupted *tlp7* reading frame [8]. Another study also showed that the presence of the interrupted reading frame is over or underrepresented in strains isolated from different sources [10]. Due to this we sequenced each *tlp7* amplicon to determine if the gene was present as a full length reading frame or if it was split into two open reading frames with the introduction of a stop codon. The PCR primers used to amplify *tlp7* were designed to amplify across the split between *Cj0951c/Cj0952c* of *C. jejuni* 11168. Sequencing data showed in 23 of the 31 strains that contain *tlp7* that it is present as an uninterrupted gene sequence (Table 1). In the remaining eight strains it exists as two separate open reading frames indicating that Tlp7 is produced as two separate proteins in these strains (Table 1).

### Relative expression of *tlp* genes by qPCR

In order to determine relative gene expression profiles of the *C. jejuni* group A *tlp* genes at varying conditions *in vitro* and *in vivo, C. jejuni* strains, 11168-GS, 11168-O and 81116 were grown *in vitro,* at 37°C, 42°C and maintained in pond water at 20–25°C, and *in vivo* by colonising avian and mammalian hosts and then isolated directly from animals by immunomagnetic separation (IMS) (Methods). Growth at 37°C, 42°C was assessed as it mimics mammalian and avian hosts *in vitro* and allows a direct comparison with expression of Tlps in cells directly isolated from animal hosts. Maintenance in pond water (from local farm pond, sterilised) at 20–25°C is used to mimic environmental conditions [12], as surface and reservoir water contamination is a potential environmental source for *C. jejuni* outbreaks [13-16]. Relative gene expression of the group A *tlp* receptors in *C. jejuni* under all these different conditions was then assessed by Quantitative PCR*.* The expression of *tlp* genes was compared between each strain and growth condition. Only statistically significant differences (p < 0.05) are described below.

### Comparison of the group A tlp gene expression for *C. jejuni* 11168-O, 11168-GS and 81116

**Figure 1:**
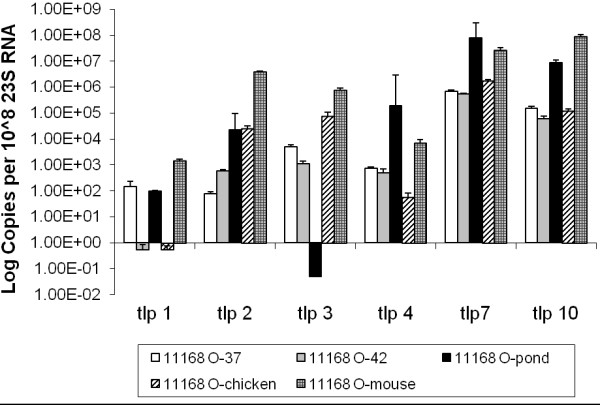
**Expression of Group A *tlp *genes for *C. jejuni *strain 11168-O.** Relative gene expression profiles of Group A *tlp* genes for *C. jejuni* 11168-O grown at 37°C, 42°C, maintained in pond water and isolated *in vivo* from chicken and mouse. Expression is standardised and the scale is shown in log (copies per 10^8^ of 23 S RNA). 37: grown under laboratory conditions at 37°C, 42: grown under laboratory conditions at 42°C, pond: maintained in an environmental water source at room temperature, 22°C, chicken: directly isolated from chicken caecal content by Dyna-beads, mouse: directly isolated from mouse intestines by Dyna-beads. Standard errors are shown as bars above the mean of a minimum of 3 independent PCR reactions.

The expression levels of *tlp* genes within *C. jejuni* strain 11168-O were generally varied, with *tlp7* and *10* showing higher expression levels compared to the other *tlp* genes. It is interesting to note that *tlp1* showed the lowest level of expression (Figure 1), particularly in cells isolated from the intestines of chicks and from bacteria grown in laboratory conditions at 42°C. Contrary to all expectations, the expression of *tlp7* was very high under all conditions tested, irrespective of the fact that it is a present as two separate gene transcripts in *C. jejuni* 11168-O (Figure 1). This high level of expression correlated with the finding that *tlp7* may act as a functional receptor even when present as two separate genes [8].

**Figure 2:**
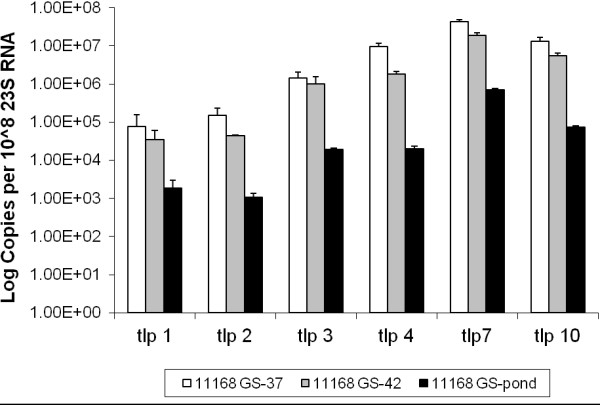
**Expression of Group A *tlp *genes for *C. jejuni *strain 11168-GS.** Relative gene expression profiles of Group A *tlp* genes for *C. jejuni* 11168-GS grown at 37°C, 42°C and maintained in pond water. Expression is standardised and the scale is shown in log (copies per 10^8^ of 23 S RNA). 37: grown under laboratory conditions at 37°C, 42: grown under laboratory conditions at 42°C, pond: maintained in an environmental water source at room temperature, 22°C. Standard errors are shown as bars above the mean of a minimum of 3 independent PCR reactions.

In contrast, the expression profiles for the group A *tlp* genes in *C. jejuni* 11168-GS all displayed similar patterns of gene expression. The expression of *tlp* genes in 11168-GS appeared to be temperature dependent with the lowest level of expression observed in bacteria maintained at room temperature in pond water (Figure 2). The highest levels of expression were observed in bacteria grown at 37°C, while in most cases expression at 42°C were lower than those seen at 37°C. Unlike *C. jejuni* 11168-O, 11168-GS *tlp* gene expression appears to be related to temperature, however not all *tlp* genes were expressed at the same level.

**Figure 3:**
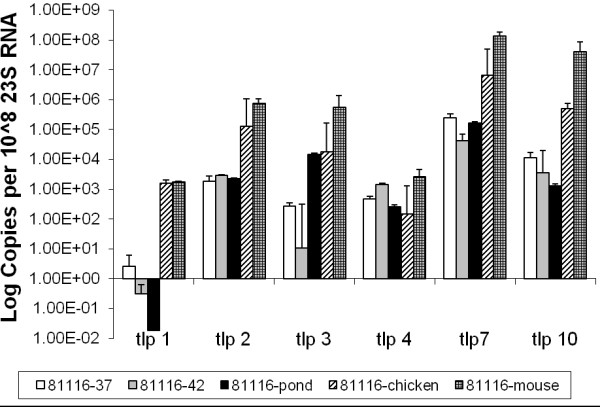
**Expression of Group A *tlp *genes for *C. jejuni *strain 81116.** Relative gene expression profiles of Group A *tlp* genes for *C. jejuni* 81116 grown at 37°C, 42°C, maintained in pond water and isolated *in vivo* from chicken and mouse. Expression is standardised and the scale is shown in log (copies per 10^8^ of 23 S RNA). 37: grown under laboratory conditions at 37°C, 42: grown under laboratory conditions at 42°C, pond: maintained in an environmental water source at room temperature, 22°C, chicken: directly isolated from chicken caecal content by Dyna-beads, mouse: directly isolated from mouse intestines by Dyna-beads. Standard errors are shown as bars above the mean of a minimum of 3 independent PCR reactions.

Gene expression profiles for the group A *tlp* genes in *C. jejuni* 81116 *in vitro* and *in vivo* were also diverse. It is notable that the expression of the aspartate receptor gene, *tlp1,* was the lowest of all *tlp* genes, with almost no detectable expression when grown at 37°C, 42°C or in pond water. In contrast, *tlp1* was highly expressed in *C. jejuni* 81116 isolated from *in vivo* hosts (p < 0.05) (Figure 3). Expression levels seen for *tlp1, tlp2, tlp3, tlp7* and *tlp10* were all higher in *C. jejuni* isolated from both *in vivo* hosts, compared to bacteria grown at an equivalent temperature under laboratory conditions, indicating that host factors are involved in stimulation of *tlp* gene expression. The expression of *tlp7* and *10* were consistently higher than the other *tlp* genes under all conditions tested, with the highest expression observed for *tlp7* in 81116 isolated from the intestines of mice.

### Verification of Tlp1 expression by Western blot

**Figure 4:**
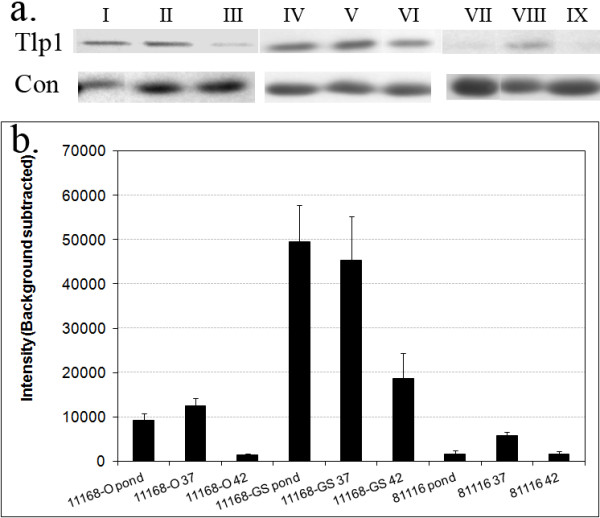
**Quantitative protein analysis of cellular Tlp1 levels. a**.) Representative blot result from a Western blot performed using anti-Tlp1 sera. Samples are as follows for Tlp1 and Con; I). *C. jejuni* 11168-O maintained at room temperature in pond water; II). grown at 37°C; III). grown at 42°C. IV). *C. jejuni* 11168-GS maintained at room temperature in pond water; V). grown at 37°C; VI). grown at 42°C. VII). *C. jejuni* 81116 maintained at room temperature in pond water; VIII). grown at 37°C; IX). grown at 42°C. A single band was observed at ~75 kDa corresponding to the predicted size of Tlp1. The loading control shows the band (~30 kDa) that was used to ensure the same amount of protein was loaded in each well. **b**.) Quantitative densitometry analysis of Tlp1 protein detected by anti-Tlp1 sera. Average background subtracted band intensity was determined using QuantityOne software (Bio-Rad) from triplicate repeat anti-Tlp1 Western blots of *C. jejuni* 11168-O, 11168-GS and 81116 maintained at room temperature in pond water; grown at 37°C; grown at 42°C. Errors bars equal to 3x standard error of the mean (SEM).

To verify that mRNA levels detected by qPCR reflected the level of protein produced in the bacterial cells, Western blot analysis was performed, using whole cell protein of *C. jejuni* 11168-O, 11168-GS and 81116 grown or maintained in the laboratory at room temperature, at 37°C and 42°C, with polyclonal antisera raised against purified periplasmic domain of Tlp1 protein (Figure 4a). Quantitative analysis of the cellular Tlp1 protein, detected by the specific antisera, showed that cellular protein levels changed according to the growth conditions. Tlp1 was present in 11168-O grown at 37°C at 1.4 fold greater than in pond water maintained bacteria, and 9.3-fold greater than in bacteria grown at 42°C (Figure 4b). These results are in agreement with qPCR analysis which showed that Tlp1 was expressed highest in *C. jejuni* grown at 37°C, 1.5-fold more than *C. jejuni* maintained in pond water at room temperature and 275-fold higher than *C. jejuni* grown at 42°C. The protein levels of Tlp1 were seen to be more than four-fold higher in *C. jejuni* 11168-GS then in any of the conditions tested for *C. jejuni* 11168-O or 81116 which correlates well with the apparent over-expression seen in 11168-GS for *tlp1*. *C. jejuni* 81116 showed the lowest protein levels also in agreement with the expression data.

## Discussion

This report describes the analysis of the group A chemosensory receptor content of various *C. jejuni* strains and the modulation of expression of the *tlp* genes under varying *in vitro* and *in vivo* conditions. Analysis of the chemoreceptor subsets demonstrated that the most conserved *tlp* genes were *tlp1* and *tlp7,* with the presence of these genes verified in all bacterial strains tested. Previous analysis of the ten sequenced strains (NCBI) revealed that in all strains, *tlp1* amino acid sequences were 99 - 100% identical [6]. It appears likely that this level of conservation is due to Tlp1 being the sensory receptor for aspartate in *C. jejuni*[7], where aspartate is one of the few carbon sources utilised in *C. jejuni* metabolism [17,18]. It is interesting to note that although *tlp1* was ubiquitously present within *C. jejuni* strains tested, it had the lowest levels of expression in all strains tested at different temperatures *in vitro* and in bacteria isolated directly from animal hosts. This observation contrasts with reports for other bacterial aspartate receptors, including Tar of *E. coli*, which is 5–10 fold more abundant than other chemoreceptors in that organism [19]. It would be interesting to determine if Tlp1 is indeed a minor receptor among others or whether there are controlling elements involved in translation and protein stability that may influence the numbers of individual receptors in receptor clusters which are yet to be demonstrated for *C. jejuni*. We can note, however, that expression of the *tlp1* gene appears to be tightly controlled for successful colonisation of chickens [7]. In Hartley-Tassell *et al.* (2009), we showed that an isogenic mutant of *tlp1* failed to properly colonise the chick model indicating that expression of *tlp1* is involved in establishing normal colonisation. We also showed that over-expression of *tlp1* was detrimental to normal colonisation as the complemented isogenic mutant of *tlp1* had comparatively higher expression levels than that seen in wild-type *C. jejuni* 11168-O and thus was only able to poorly complement the mutant [7].

Similar to the aspartate sensory receptor, *tlp7* was present in 31 of the 33 strains tested in this study. *Tlp7* was previously reported as being a “pseudogene” in *C. jejuni* 11168 [5] and in all but one of the sequenced strains (NCBI), *C. jejuni* HB93-13 [6]. However, with the full annotated sequence of *C. jejuni* 81116 and an updated annotation of *C. jejuni* 81*–*176 being released, *tlp7* has been reassigned as a functional gene in these strains, which agrees with our sequence analysis. Interestingly, *tlp7* shows amino acid identity of >93% among the strains we tested, irrespective whether the gene was an uninterrupted open reading frame or if it was present as two open reading frames separated by a stop codon. In addition, *tlp7* was highly expressed, often being the most abundantly expressed of all group A *tlp* genes in strains 81116 and NCTC 11168 (both -GS and –O) which were tested using different growth conditions, including expression *in vivo* in murine and avian hosts. It has been shown that the two proteins of Tlp7, *Cj0951c* and *Cj0952c*, are expressed separately but can still function as a formic acid receptor [8]. This indicates that the periplasmic and cytoplasmic domains of Tlp7 encoded by *Cj0951c/Cj0952c* are likely to be able to integrate into sensory receptor clusters and interact in order to transduce the signal to the CheAY/CheW/CheV complex [7,8].

The second most commonly occurring chemoreceptors were *tlp3* and *tlp10. Tlp3* was absent in 81–176, 331 and GCH11 but showed highly variable expression depending on the strain of bacteria and the growth/maintenance condition tested. Expression of *tlp10* was high in all strains at most of the conditions tested. Although no ligand has been identified for Tlp10 in *C. jejuni,* the high overall expression of *tlp10* indicates that the ligand may be of some importance for the survival or colonisation of this organism. Expression of this receptor gene within an animal host, in particular the murine model, was higher than under laboratory conditions, with 100–1000 fold greater expression in mice than in chickens indicating a possible role of *tlp10* in opportunistic infection of mammalian hosts.

The presence of *tlp2* and *4* within the genomes of *C. jejuni* were the most variable with 13 strains lacking one or both of these genes. This result is comparable to the analysis of the sequenced strains of *C. jejuni* (NCBI) with four of the 10 strains lacking one or both of *tlp2* or *4*. Like Tlp3, the amino acid sequences of Tlp2 and 4 are less conserved than Tlp1 and 10. The expression levels of *tlp2* and *tlp4* were variable between strains and conditions tested with *tlp2* being one of the most abundantly expressed *tlps* in *C. jejuni* 11168-O isolated from mice. Little is known about either Tlp2 or Tlp4 with respect to ligand binding specificity; however it is interesting to note that these two Tlps along with Tlp3 share almost 100% homology within the cytoplasmic signalling domain of the proteins [5].

Interestingly one of the recently acquired hospital isolates, GCH11, lacked all three of these *tlps* (*tlp2, 3* and *4*). This strain only possessed *tlp1, 7*^*w*^*, 10* and *11* and was able to produce disease of sufficient severity to require hospitalisation. While no data is available on the age or immune competency of the patient, it is clear that a strain with this subset of receptors is able to efficiently infect a human host and cause disease. In 11168-O and 81116, *tlp1, 7* and *10* were all induced when in an animal host as compared to laboratory growth conditions. The regulation of *tlp11* under host conditions is currently unknown.

Tlp11 was the least common of the group A *tlps*, only present in the genome of ten of the 33 strains tested and only found in one of the 10 sequenced strains of *C. jejuni,* 84*–*25. The expression of *tlp11* did not vary with the conditions tested. As yet the ligand for Tlp11 is unknown but interestingly *C. jejuni* 84–25 is an isolate from a rare *Campylobacter* meningitis case [20], while 520 is a highly invasive strain of *C. jejuni*[6] and each of the Gold Coast Hospital isolates were of sufficient disease severity that the infected individuals required hospitalisation. Thus suggesting that Tlp11 may in fact be a marker of virulence in *C. jejuni*.

It is important to note that *C. jejuni* 11168-GS and 11168-O express group A *tlp* genes differently under the same conditions, with 11168-GS generally expressing the *tlps* at a higher and more uniform level than 11168-O. A representative example of this difference was the expression of *tlp1* at growth temperatures of 37°C and 42°C with *C. jejuni* 11168-GS expressing *tlp1* up to 10,000 fold greater than 11168-O. The protein level of Tlp1 in *C. jejuni* 11168-GS was also shown to be significantly higher than that seen for 11168-O. Gaynor *et al.* (2004) noted changes in sigma factor sequences indicating that there may be changes in the way genes are regulated in the two strains [11]. While their analysis did not discover any expression changes in *tlps* there was expression changes in genes involved in chemotaxis, such as CheW and flagella. In addition, there were differences noted in amino acid uptake and catabolism genes including some involved in the processing of aspartate [11]. The comparison of data presented here and that already shown by Gaynor *et al.* (2004) indicates that there is likely to be a broad disregulation of chemotaxis and the processing of the molecules that are known to be ligands for *C. jejuni* chemotaxis in 11168-GS. This disregulation may be directly related to the protein sequence changes noted in the three sigma factors screened [11]. As we have previously mentioned, tight control of *tlp1* expression appears to be important for optimum colonisation of chickens [7]. It is therefore possible to speculate that the altered expression of *tlps* in 11168-GS may contribute to reduced ability of this variant to colonise animals and to invade mammalian cells in cell culture [11].

## Conclusion

In conclusion, this study has demonstrated that chemoreceptor subsets vary between *C. jejuni* strains with the aspartate receptor, *tlp1,* conserved in all subsets observed. Expression of chemosensory group A *tlp* genes was similar between strains with *tlp7* and *tlp10* typically the highest expressed *tlps* and with expression generally higher in animal hosts than under laboratory conditions.

## Methods

### *C. jejuni* strains and growth conditions

*C. jejuni* strains NCTC 11168-GS, 11168-O (original Skirrow’s isolate) and 81116 were kindly donated by D.G Newell (Veterinary Laboratory Agency, London, UK). Human isolates 173, 351, 430, 435, 440, 520, 705, 8, 193 and chicken isolates 019, 108,331, 434, 506, 008 and 193 were from RMIT/Griffith Universities culture collections, *C. jejuni* 81–176 was kindly donated by J. Fox, MIT, Boston, USA and *C. jejuni* GCH1-17 were collected between 19/01/2010 and 12/03/10 by S.K. Day from Queensland Health Pathology, Gold Coast Hospital, Queensland, Australia. *Campylobacter* cells were grown on solid selective agar (Columbia agar, 5% (v/v) defibrinated horse blood, Skirrow Selective Supplement; Oxiod) under microaerobic conditions (5% O_2_, 15% CO_2_, 80% N_2_; BOC gases) for 48 hours at 42°C. *C. jejuni* was harvested from the agar plates in sterile Brucella Broth (BBL) and the cfu/mL was determined by measuring OD_600nm_ and comparing to a standard growth curve. Cultures for RNA analysis were grown under the following conditions: Cultures that mimic environmental conditions were performed as previously described [12]. Cultures grown for laboratory conditions were grown at either 37 or 42°C as described in Day *et al.* (2009) and processed to minimise effects on RNA expression as per King *et al.* (2012) [12,21].

### PCR amplification of *C. jejuni* Group A *tlp* genes

A single 10 μL loop full of bacteria was removed from an agar plate covered with a confluent growth of the *C. jejuni* strains and resuspended in 1 mL of sterile water. The bacterial suspension was boiled for 5 min and the cell debris was pelleted by centrifugation at 13,000 g, and the supernatant was used as template DNA for PCR analysis. The template DNA was used at 10% of the final PCR volume in the presence of 10 ρmoles of forward and reverse primer (Table 2), 10 μM dNTPs, 1x polymerase reaction buffer, 1 unit of thermal stable DNA polymerase and 3.5 mM MgCl_2_. The PCR reaction was performed as follows; 95°C for 5 mins for 1 repeat, 95°C for 30 seconds, 50°C for 1 minute and 72°C for 1 minute for 45 repeat cycles followed by a final extension of 72°C for 5 minutes. Presence of PCR product amplification was determined by agarose gel electrophoresis.

**Table 2:** Primers used in this study

**Primer name**	**5`-3` primer sequence**
Tlp1p F	TTG TTA TCG TTT ACG CTG ATG
Tlp1p R	TGG AAG ATC TTT ATT ATA ATT TTT TAA GGG TTT AA
Tlp2p F	CAT ATG CAA GCA ATT TTT CAT GAA GTT GTG A
Tlp2p R	CTC GAG TTA TTT ATA AAC TGG AGC TTC TAT TTG TT
Tlp3p F	CAT ATG ACC TCA CTA TAT GAA AGC ACT CTT
Tlp3p R	CTC GAG TTA TGC AGC TTT ATA AAT AGG TTT ATT TAT A
Tlp4p F	CTC GAG GAT TCG AGA AAC AAT ACA TAT GAA TT
Tlp4p R	CTC GAG TTA TTG TTT CAT TAA AAT AGA ATT AAC AGC
Tlp7p F	CAT AGT TTT AAA AAT ACT GCC AAT AAA ATG AG
Tlp7p R	CTC GAG TTA AGA TTG ACT GGT TTT GCT TAT ATC
Tlp7i F	CTG CGA TCT CAT CCA TCA TTT GAG TTG C
Tlp7i R	CAT GCT AAA GAA TTA GCT CAA GGA AGT GGC
Tlp10p F	CAT ATG AAC TAT TCT TCA TCT AAA GAT AAT AA
Tlp10p R	CTC GAG TTA TTT AAA TAA ATT AGA TTG TTC TAT AGT
Tlp11mid F	CTC TGA TGG CAA AAG TGT AAC
Tlp11mid R	CTC TTC AGA TTG AGC GAT AAC
Therm 1 (23SRNA)	TTA TCC AAT ACC AAC ATT AGT
Therm 2.1 (23SRNA)	GAA GAT ACG GTG CTA TTT TG

### Preparation of *C. jejuni* inoculum

*C. jejuni* cells were harvested from Columbia agar plates in 1 mL of PBS (137 mM NaCl, 2.7 mM KCl, 10 mM Na_2_HPO_4_ and 1.8 mM KH_2_PO_4_, pH 7.5) and the concentration was adjusted to 1 x 10^8^ cfu/mL using spectrophotometry followed by a viable count.

### Inoculation of chickens with *C. jejuni*

Ross breed chickens (Barters, Rochdale, Qld), with maximum age difference of 2 hours and at one day after hatching, were placed into groups of five, colour marked and pre-inoculation faecal samples were taken from the cloaca and cultured. Chickens were housed in clean barrier cages at 28°C and allowed access to sterilised food and water. All experiments were approved by the Griffith University Animal Ethics Committee (approval number: MSC/04/08/AEC).

Following a pre-inoculation cloacal swab, one day old chickens were orally inoculated with 30 μL PBS containing 1 x 10^8^ cfu bacterial cells as previously described [22]. On day 6, euthanasia was performed by cervical dislocation. Post-mortem caecal samples were obtained by the dissection of the caeca aseptically. Whole *C. jejuni* cells were collected directly from the caeca with the use of antibody coated M-280 Dyna-beads as previously described [21].

### Inoculation of mice with *C. jejuni*

Murine studies were performed using the 129X1/SvJ background male mice (Animal Resource Centre, Western Australia) aged between 6–8 weeks as previously described [23]. The mice were housed under clean conventional conditions in groups of 4–6, with free access to sterilised food and water. All experiments were approved by the Griffith University Animal Ethics Committee (Approval number: BDD/01/07).

Following a pre-inoculation swab, 129X1/SvJ mice were orally inoculated with 30 μL PBS containing 1 x 10^8^ cfu of bacterial cells. After 48 hour post-inoculation, the animals were euthanised by cervical dislocation, and the gastrointestinal tissues, small and large intestine, were collected aseptically [23]. The contents of the intestines were removed and whole *C. jejuni* cells were isolated directly from the sample with the use of antibody coated M-280 Dyna-beads as previously described [21].

### Immunomagnetic separation (IMS) of *C. jejuni* from chicken and mouse intestinal content

Immunomagnetic separation (IMS) of *C. jejuni* from chicken and mouse intestinal content was performed as previously described [21]. Briefly, intestinal content or caecal content was removed and Brucella Broth was added to a final volume of 2 mL. After removal of debris, 80 μL of anti-*C. jejuni* (Fitzgerald) coated M-280 Dyna-beads were added to the intestinal or caecal content and incubated with tilt rotation at 4°C for 30 mins. Dyna-beads were removed from the sample using IMS and washed three times with Isotonic PBS containing 0.1% tween-20 at 4°C. Bound *Campylobacter* was eluted from the beads using 0.05% trypsin-EDTA (Invitrogen), supernatant was removed and centrifuged at 10,000 x *g* to yield a bacterial pellet. RNA was extracted using Qiagen RNase easy kit, with on-column DNase digestion.

### Primer design

Primers were designed based on the published nucleotide sequence of *C. jejuni* 11168 [24] to allow PCR amplification of the periplasmic sensory domain of the group A *tlp* receptors, *tlp1-4, 7* and *10*. *Tlp11* primers were designed on the sequence of *tlp11* from *C. jejuni* 520 (sequence not published) and the sequenced strain 84–25. Therm 1 and 2.1 primers, which amplify the 23 s RNA gene [25] were used as internal control. Primers used in this study are listed in Table 2.

### Q RT-PCR analysis of *tlp* expression in *C. jejuni*

Total RNA was extracted using RNeasy kit according to manufacturer’s protocol (Qiagen) with on-column DNase. Extracted RNA was used as template for the reverse transcription reaction; 10 μL of cDNA was synthesised by using gene specific primers (Table 2) and Improm II reverse transcriptase (Promega). All samples were reverse transcribed under the same conditions, 42°C for 1 hour, and the same reverse transcriptase mastermix, to reduce differences in RT efficiency.

Q RT-PCR was performed in 20 μL with 1.5 μL of cDNA, 10 μL Sensimix (Quantace) and 250 nM sense and anti-sense primers (Table 2). The qPCR reactions were carried out using a Bio-Rad iQ5 PCR machine and Bio-Rad iQ5 optical system software program.

All qPCR reactions were carried out using the same thermal profile conditions, 94°C for 5 minutes, then 45 cycles of 94°C for 30 seconds, 48°C for 30 seconds then 72°C for 1 minute, 30 seconds with fluorescence measured during the 72°C extension phase. Melt curves were produced for each amplification product and these were measured 80 times over the incremental increases in temperature. Amplification plots and melt curves were analysed by the Bio-Rad iQ5 optical system software program. Products were reconfirmed by performing agarose gel electrophoresis.

A PCR standard curve was generated for each primer set by performing five ten-fold serial dilutions. Quantity values (copies) for gene expression was generated by comparison of the fluorescence generated by each sample with a standard curve of known quantities for each PCR product. The standard curve equations are listed in Table 3.

**Table 3:** PCR standard curves

**Gene**	**standard curve equation**	**efficiency**
Tlp1	y = −3.764 + 42.062	84.3%
Tlp2	y = −3.670 + 37.969	95%
Tlp3	y = −3.638 + 43.558	88%
Tlp4	y = −2.288 + 34.017	173%
Tlp7	y = −3.486 + 45.126	93.6%
Tlp10	y = −3.641 + 45.241	88.2%
Tlp11	y = −5.297 + 60.289	54.4%
23 S RNA	y = −3.828 + 43.454	82.1%

### Immunisation of mice and production of polyclonal anti-sera

Preimmune serum was collected prior to immunisation and tested for reactivity with *C. jejuni* and with purified Tlp1 protein. Five female BALB/c mice (SPF) were injected subcutaneously with a total volume of 200 μL consisting of 50 μg of His-tagged Tlp1^peri^, expressed and purified as previously described [7], combined with an equal volume of Freund’s Incomplete adjuvant (Sigma) on day 0. On days 14, 28 and 42 mice were boosted subcutaneously with 25 μg of His-tagged-Tlp1^peri^ combined with an equal volume of Freund’s incomplete adjuvant (Sigma). A test-bleed was taken on day 35. On day 56, blood was harvested via cardiac puncture. Blood was allowed to clot at room temperature and the serum was collected for further use. The specificity of anti-Tlp1^peri^ serum was verified by Western blot analysis and ELISA against cell lysates. All experiments were approved by the Griffith University Animal Ethics Committee (Approval number: BDD/01/09).

### Western blot analysis of Tlp1

*C. jejuni* lysates of bacteria grown or maintained at room temperature, 37°C and 42°C were prepared by the harvesting of 10^9^ bacteria per mL in autoclaved water. 40μL of this suspension (4x10^7^*C. jejuni*) were mixed with SDS-PAGE loading buffer and boiled for 5 minutes and loaded onto the gel. SDS-PAGE and Western blot were performed as previously described [26] using a 1:200 dilution of the anti-Tlp1^peri^ serum. Cell counts were verified to ensure equal number of bacteria was used in each well. Reactivity of the anti-sera to specific antigens was detected as previously described [7]. An anti-*C. jejuni* antibody (Fitzgerald) was also used to obtain a loading control. Briefly, the anti-*C. jejuni* antibody was used to screen blots of whole *C. jejuni* (4x10^7^) maintained under the conditions listed above. All of the proteins bound by the antibody were analysed using QuantityOne software (Bio-Rad) to identify the one with the least variability between conditions and strains. A ~30 kDa protein was identified as the least variable with no significant change detected in expression between strains or growth conditions. This band was then used for the loading controls.

## Abbreviations

Tlp, Transducer Like Protein; IMS, ImmunoMagnetic Separation.

## Competing interests

The authors declare that they have no competing interests.

## Authors’ contribution

CJD Performed and planned experiments and wrote large portions of the final manuscript. LEHT Performed and planned experiments and wrote large portions of the final manuscript. LKS Produced antibody for analysis of Tlp1 and performed experiments utilising this antibody. Also helped in the preparation of the final manuscript. RMK Helped plan and performed animal work and helped prepare the final manuscript. GT Performed and planned many of the experiments involving Tlp11 and helped prepare the final manuscript. SKD Identified, isolated and provided fresh clinical isolates for this publication. EAS Helped perform animal work and preparation and performing of experiments involving GCH isolates and aided in the preparation of the final manuscript. VK Devising of initial experiment, planning of experiments and drafting of the manuscript. All authors read and approved the final manuscript.
